# High-cervical solitary fibrous tumour—a case report, and mini-bibliometric analysis

**DOI:** 10.1093/jscr/rjaf492

**Published:** 2025-07-11

**Authors:** Yong Yie Liew, Zoubeyr Abbou, Tobechi Mbadugha, Zuhair Karmi

**Affiliations:** School of Medicine & Dentistry, University of Central Lancashire, Fylde Rd, Preston, Lancashire, PR12HE, United Kingdom; Department of Neurosurgery, Royal Preston Hospital, Sharoe Green Lane North, Fulwood, Preston, Lancashire PR29HT, United Kingdom; Department of Neurosurgery, Royal Preston Hospital, Sharoe Green Lane North, Fulwood, Preston, Lancashire PR29HT, United Kingdom; Department of Neurosurgery, Royal Preston Hospital, Sharoe Green Lane North, Fulwood, Preston, Lancashire PR29HT, United Kingdom

**Keywords:** solitary fibrous tumour, spinal cord, diagnosis, management, follow-up

## Abstract

We present a case report of a 53-year-old left-handed gentleman who presented with 8 months of right-sided neck pain and radiating arm pain. MRI spine showed suspicion of an intradural, extramedullary lesion initially thought to be a schwannoma. Single-level laminectomy and resection was carried out and noticeable residuum cauterized. Histopathology showed World Health Organization Grade I solitary fibrous tumour (SFT) with Ki-67 of 6%. No major complications post-operatively. Follow-up MRI spine showed gross total resection in the spinal canal with small residual arising from the nerve root. Solitary fibrous tumour are rare mesenchymal tumour especially in the cervical spine. Higher World Health Organization grade and subtotal resection has been associated with higher recurrence rate and metastases. No high-quality evidence available comparing different standards of treatment and follow-up protocol/treatment. Hence, no consensus available with regards to the optimal management, adjuvant therapy, and follow-up protocol. But regular follow-ups recommended especially for higher-grade tumours.

## Introduction

Solitary fibrous tumour (SFT) are spindle-cell neoplasms of mesenchymal, non-meningothelial origin, and remained a rare central nervous system (CNS)-occurring lesion, more so within the spine, up to 13% of known CNS SFT [1]. The term ‘hemangiopericytoma’ and ‘solitary fibrous tumour/hemangiopericytoma’ has been removed from the World Health Organization (WHO) 2021 CNS classification which align with the soft tissue nomenclature. It is associated with NAB-2-STAT6 gene fusion. WHO 2021 has graded SFT into three different grades ranging from I (lowest) to III (highest) [2].

Radiological findings on MRI are not conclusive for SFT and can be easily mistaken for schwannomas and meningiomas which are more common epidemiologically. The most common spinal level identified is thoracic, followed by cervical and lumbar [3].

We present our case report of high-cervical SFT and mini-bibliometric analysis of the current literature.

## Case report

A 53-year-old left-handed gentleman with known background of hypothyroidism and previous stroke was referred to the neurosurgical service with 8 months history of right neck pain and right arm pain (Pain score of 8/10). He also describes progressive numbness, loss of proprioceptive sensations in his right arm, and progressive weakness. Has been having more frequent falls as he described legs giving away. He denies bladder or bowel dysfunction.

On examination, he is hyperreflexia and hypertonic in all limbs with positive Hoffmann’s and Babinski sign bilaterally, reduced sensation in all limbs of no specified dermatomal patterns, and loss of proprioceptive sense in his right arm. Motor examination described in Table 1a and b.

**Table 1a:** Motor examinations for the upper limbs pre-operatively.

Movements	Right upper limb	Left upper limb
Shoulder abduction	3/5	5/5
Shoulder adduction	3/5	5/5
Elbow flexion	4/5	4+/5
Elbow extension	4/5	4+/5
Wrist flexion	3/5	4+/5
Wrist extension	3/5	4+/5
Finger flexion	3/5	5/5

**Table 1b:** Motor examinations for the lower limbs pre-operatively.

Movements	Right lower limb	Left lower limb
Hip flexion	4/5	5/5
Hip extension	4/5	5/5
Knee flexion	4/5	5/5
Knee extension	4/5	5/5
Ankle dorsiflexion	4/5	5/5
Ankle plantarflexion	4/5	5/5
Extensor hallucis longus	5/5	5/5

Bloods were unremarkable with inflammatory markers (white blood cells and C-reactive protein) within normal ranges.

**Figure 1:**
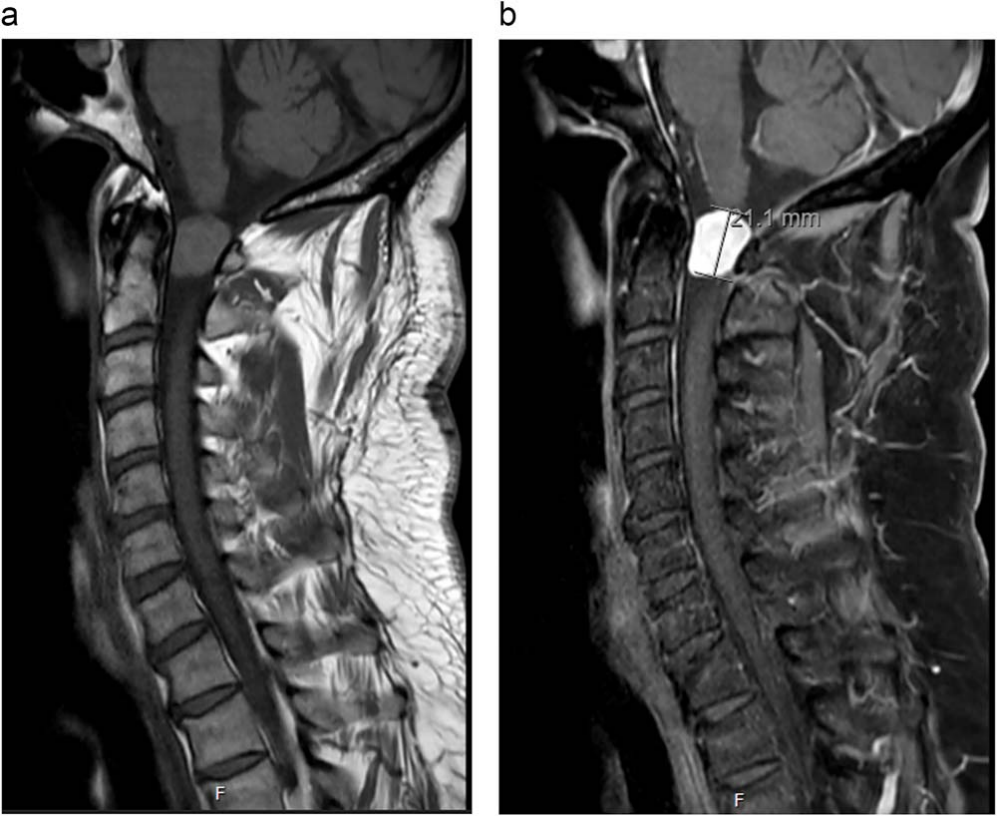
(a) Pre-operative sagittal MRI cervical spine T1-weighted image without contrast. (b) Pre-operative sagittal MRI cervical spine T1-weighted image with contrast.

**Figure 2:**
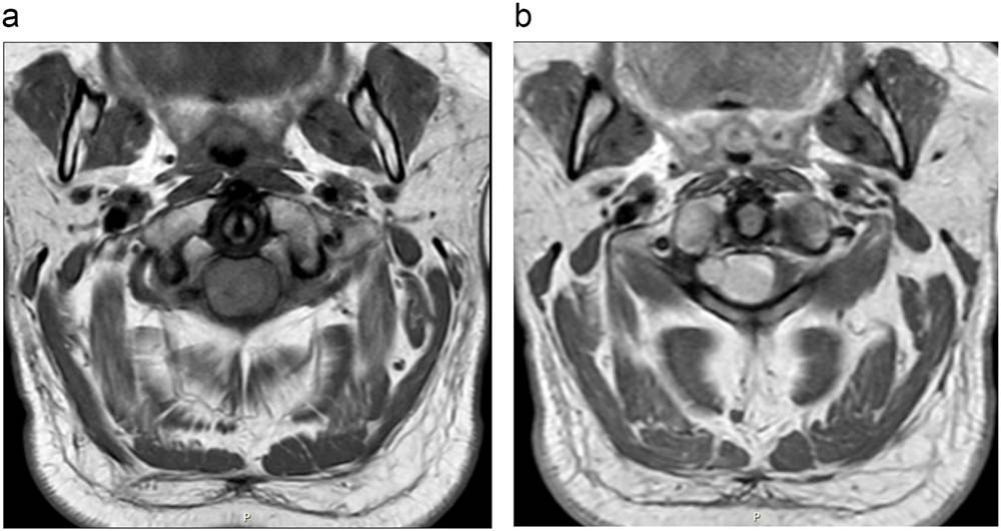
(a) Pre-operative axial MRI cervical spine T1-weighted image without contrast (across the C1 level). (b) Pre-operative axial MRI cervical spine T1-weighted image with contrast (across the C1 level).

MRI cervical spine (Fig. 1) demonstrated an extramedullary intradural lesion at C1/2 within the right aspect of the canal distorting and compressing the cord to the left with associated cord signal changes. The lesion is isointense on T1- and T2-weighted images and measures ~1.7 × 2.0 × 2.1 cm. Following contrast (Fig. 2), there is homogenous enhancement of the lesion. Computed tomography of thorax, abdomen, and pelvis showed no evidence of primary lesions or skeletal deposits.

Case was discussed on complex spine Multidisciplinary Team with the consensus that appearances in keeping with schwannoma and planned for surgical resection. C1-laminectomy and excision of the lesion was carried out under general anaesthetic. Whole tumour was removed apart from small tumour arising from the right C2 nerved root as it was adherent to the nerve root. Residual tumour arising from the nerve root was cauterized.

Frozen section demonstrated a poorly differentiated tumour composed of tightly packed cells with pleomorphic nuclei and minimal cytoplasm. No clear features of meningothelial or schwannian differentiation present in the tissue. Microscopically, the tissue demonstrated tumour cells with spindled to ovoid nuclei and single nucleoli and no evidence of necrosis. A number of dilated ‘staghorn’ vessels noted within the tumour. Mitotic activity was sparse and does not exceed 2 mitoses per 10-high-power-field. Immunohistochemistry demonstrated diffuse staining of CD34, nuclear labelling of STAT6, weak patchy staining of EMA, no staining of SSTR2a, no staining of S100, no staining of AE1/3, and ~6% of Ki-67. Final diagnosis of WHO Grade I SFT was made. Post-operatively, he remained well with no major post-operative complications, no change in his neurological findings and was discharge 6 days after.

Post-operative MRI done 6 weeks following the procedure demonstrated near total resection of the intradural extramedullary tumour with small component of tumour seen along the exiting right C2 nerve root. There is complete resolution of mass effect of the spinal cord at C2 level.

He was reviewed at 8 and 12 weeks following discharge. Clinically, the power of his limbs have improved to 5/5 except for right shoulder abduction which was 4+/5 with improvement in his upper limb sensations. No post-operative adjuvant therapy was offered due to low WHO grade following discussion with the oncology team, but patient was placed on regular follow-ups.

### Mini-bibliometric analysis

We conducted a mini-bibliometric analysis on Web of Science using the search strategy presented in Supplementary File 1. Thirty-four articles (Table 2) were included with publication year ranges from 1996 to 2024. The highest citation is 95 and lowest of 0. Four articles described cervical SFT metastasis, while 28 articles described primary cervical SFT. Most are case reports with/without literature review (*n* = 26), 7 observational studies, and 1 literature review. Total number of patients (excluding literature review) identified were 60. No randomized controlled trials or systematic review/meta-analysis focusing on cervical SFT identified.

**Table 2:** Bibliometric analysis of articles included relating to cervical spine SFT from highest total citations to lowest total citations

Title	Year of publication	Total number of citations (average yearly citations)	Primary/cervical metastases	Number of patient(s) with cervical disease
Solitary fibrous tumour of the central nervous system: A 15-year Literature Survey of 220 cases (August 1996–July 2011)	2011	95 (6.33)	Not applicable (purely literature review)	Not applicable (purely literature review)
The central nervous system solitary fibrous tumour: A review of clinical, imaging, and pathologic findings among all reported cases from 1996 to 2010	2011	65 (4.33)	Not stated	16
Solitary fibrous tumour of the spinal nerve rootlet: Case report and literature survey	1999	34 (1.26)	Primary	1
Solitary fibrous tumour of the spinal cord	2000	30 (1.15)	Primary	1
Intramedullary and extramedullary solitary fibrous tumour of the cervical spine—Case report and review of the literature	2004	29 (1.32)	Primary	1
Spinal solitary fibrous tumour/hemangiopericytoma: a clinicopathologic and radiologic analysis of 11 cases	2017	23 (2.56)	Primary	2
Spinal hemangiopericytoma: an institutional experience and review of literature	2015	20 (1.82)	Primary	2
Primary hemangiopericytoma in the axis bone: Case report and review of literature	1996	20 (0.67)	Primary	1
Hemangiopericytoma of the cervicothoracic spine: a case report and literature review	2014	17 (1.42)	Primary	1
Solitary cervical fibrous tumour—case illustration	2003	16 (0.7)	Primary	1
Solitary fibrous tumour in the cervical spine with destructive vertebral involvement: a case report and review of the literature	2008	15 (0.83)	Primary	1
Hemangiopericytoma-like synovial sarcoma of the lumbar spine—Case report	2006	15 (0.75)	Metastasis	1
Solitary fibrous tumours of the spine: a paediatric case report with a comprehensive review of the literature	2017	14 (1.56)	Primary	1
Spinal metastasis from cranial meningeal hemangiopericytomas	2006	14 (0.7)	Metastasis	1
Cervical intra-/extramedullary solitary fibrous tumour	2005	12 (0.57)	Primary	1
Intramedullary solitary fibrous tumour of cervicothoracic spinal cord	2014	11 (0.92)	Primary	1
A dumbbell-shaped solitary fibrous tumour of the cervical spinal cord	2008	11 (0.61)	Primary	1
An institutional review of 10 cases of spinal hemangiopericytoma/solitary fibrous tumour	2020	9 (1.5)	Primary	4
Hemangiopericytoma/solitary fibrous tumour in the central nervous system. Experience with surgery and radiotherapy as a complementary treatment: A 10-year analysis of a heterogeneous series in a single tertiary centre	2020	9 (1.5)	Primary	3
Recurrence of solitary fibrous tumour of the cervical spinal cord	2014	9 (0.75)	Primary	1
Myopericytoma at the craniocervical junction: clinicopathological report and review of a rare perivascular neoplasm	2019	7 (1)	Primary	1
Solitary fibrous tumour of the spine: imaging features of a commonly misdiagnosed entity	2018	6 (0.75)	Primary	5
Distinctive characteristic features of intramedullary hemangiopericytomas	2015	6 (0.55)	Primary	2
Solitary fibrous tumour with multiple intracranial and spinal lesions: case report	2011	6 (0.4)	Metastasis	1
Primary endodermal hemangiopericytoma/solitary fibrous tumour of the cervical spine: a case report and literature review	2021	5 (1)	Primary	1
Spinal solitary fibrous tumour of the neck: Next-generation sequencing-based analysis of genomic aberrations	2020	4 (0.67)	Primary	1
Microsurgical resection of cervical intradural juxtamedullary solitary fibrous tumour: two-dimensional operative video	2020	2 (0.33)	Primary	1
Delayed cervical spine metastasis from intracranial solitary fibrous tumour	2023	1 (0.33)	Metastasis	1
Solitary fibrous tumour of cervical spinal cord	2020	1 (0.17)	Primary	1
Subarachnoid disseminative hemangiopericytoma of the spinal cord	2010	1 (0.06)	Primary	1
Challenges in the management of recurrent CNS solitary fibrous tumours: a case report	2024	0 (0)	Primary	1
Combined intramedullary and intradural extramedullary solitary fibrous tumour in cervical spine	2023	0 (0)	Primary	1
Cervical intramedullary solitary fibrous tumour	2024	0 (0)	Primary	1
Recurrent solitary fibrous tumour of the spinal cord: a case report and literature review	2020	0 (0)	Primary	1

## Discussion

There are currently no high-quality evidence to support the type of primary management and follow-up protocol/treatment. But most cases are treated surgically with the aim to achieve gross total resection (GTR). Recurrent disease is strongly linked to higher WHO grade and subtotal resection (STR). Metastases is linked to higher WHO grade (typically Grades II and III) and also recurrent disease. Factors such as age, gender, tumour location, and initial tumour size are not significantly associated with recurrence and metastases [4–6]. The average time to recurrence was found to be 5.7 years. The recurrence after GTR is roughly 19% and 65% after STR after 2.5 years [3]. Average time for metastases formation is significantly shorter for spinal SFT compared to intracranial (5.1 vs. 8.7 years). The overall survival rate was reported to be roughly 84% at 5 years for GTR compared to 38% in STR [4].

When GTR has been achieved, about 45% of cases reported to offer adjuvant radiotherapy (RT) and up to 85% was offered RT when STR was achieved [4]. Adjuvant chemotherapy was offered in 5%–11% of known reported cases [3, 7]. Use of adjuvant RT remained controversial with contradicting results [1, 8], but recent systematic review and meta-analyses has shown that it was associated with better recurrence control especially with higher-grade tumour, but has not shown significant impact on metastases formation and improvement to overall survival [4, 7–9].

## Conclusion

Due to the sparsity of high-quality literature, management of spinal SFT remained controversial but surgical resection tends to be the primary option with aim to achieve GTR. Regular follow-up is recommended with or without adjuvant therapy due to the possibility of recurrence and metastases especially for higher WHO grade tumours.

## Supplementary Material

Supplementary_File_1_rjaf492

## Finding

None declared.
