# Supplementary material for: High-cervical solitary fibrous tumour—a case report, and mini-bibliometric analysis
Source: J Surg Case Rep. 2025 Jul 11;2025(7):rjaf492. doi: 10.1093/jscr/rjaf492 (PMC12253951; doi:10.1093/jscr/rjaf492)
Supplement: Supplementary_File_1_rjaf492 [file supplementary_file_1_rjaf492.docx]

**Supplementary File 1**

**Database:** Clarivate™ (Web of Science™). © Clarivate 2025

**Search strategy:**

[Author keywords] (Solitary Fibrous Tumour OR Solitary Fibrous Tumor OR Hemangiopericytoma OR Hemangiopericytomas OR Solitary Fibrous Tumour/Hemangiopericytoma)

AND

[Topic] (spine OR spinal OR vertebrae)

AND

[Topic] (Cervical)

**Aim of search:**

1. To identify published literature relating to solitary fibrous tumour involving the cervical spine.
2. To identify the type of publications and the number of patients reported with the cervical SFT (primary/metastases)

**Date of search:** 2/5/2025

**Dates included:** From inception to 2025

**Sorted by:** Citations: Highest first

**Inclusion criteria:** Any articles which relates to solitary fibrous tumour involving the cervical spine regardless of primary disease or metastasis.

**Exclusion criteria:** If the report stated it is not a solitary fibrous tumour or it does not involve the cervical spine.

**Number of articles obtained on initial search:** 41 articles

**Articles removed:**

- Six articles removed as the SFT did not involve the cervical region [1, 2, 3, 5, 6, 7]
- one article removed as it is not SFT [4]

**Articles which has been removed:**

1. Kumar R, Wani AA. Unusual tumors of the posterior fossa skull base. Skull Base. 2006 May;16(2):75-84. doi: 10.1055/s-2006-934103. PMID: 17077871; PMCID: PMC1502038.
2. Tomek M, Bravi I, Mendoza N, Alsafi A, Mehta A, Molinaro L, et al. Spinal extradural solitary fibrous tumor with retiform and papillary features. Ann Diagn Pathol. 2013 Jun;17(3):281-7. doi: 10.1016/j.anndiagpath.2013.01.002. Epub 2013 Feb 8. PMID: 23632234.
3. Li Z, Deng Y, Li Z, Wang T, Gao J, Zhou W, et al. Primary epidural hemangiopericytoma of the thoracic spine: Case report and literature review. J Clin Neurosci. 2019 Feb;60:142-147. doi: 10.1016/j.jocn.2018.10.057. Epub 2018 Oct 21. PMID: 30352760.
4. Kwon JH, Song JS, Jung HW, Lee JS, Cho KJ. Malignant Solitary Fibrous Tumor with Heterologous Rhabdomyosarcomatous Differentiation: A Case Report. J Pathol Transl Med. 2017 Mar;51(2):171-175. doi: 10.4132/jptm.2016.08.29. Epub 2017 Feb 3. PMID: 28152587; PMCID: PMC5357753.
5. Fiorenza V, Ascanio F, Ferlito F, Lo Duca B. Primary intra and extradural solitary fibrous tumor/hemangiopericytoma of thoracic spine with paravertebral intrathoracic spread: Case report and review of the literature. Interdisciplinary Neurosurgery 21(1):100746. DOI:[10.1016/j.inat.2020.100746](http://dx.doi.org/10.1016/j.inat.2020.100746)
6. Mashaly H, Zhang Z, Shaw A, Youssef P, Mendel E. Intraoperative Transpedicular Onyx Injection to Reduce Vascularity of a Thoracic Hemangiopericytoma After Unsuccessful Preoperative Endovascular Embolization: a Technical Report. Oper Neurosurg (Hagerstown). 2018 Feb 1;14(2):E17-E22. doi: 10.1093/ons/opx102. PMID: 29351687.
7. Zhang YW, Xiao Q, Zeng JH, Deng L. Solitary Fibrous Tumor of the Lumbar Spine Resembling Schwannoma: Case Report and Review of the Literature. World Neurosurg. 2019 Apr;124:121-124. doi: 10.1016/j.wneu.2019.01.004. Epub 2019 Jan 14. PMID: 30654157.
